# RIG-I Is Required for the Inhibition of Measles Virus by Retinoids

**DOI:** 10.1371/journal.pone.0022323

**Published:** 2011-07-19

**Authors:** Kaitlin J. Soye, Claire Trottier, Chris D. Richardson, Brian J. Ward, Wilson H. Miller

**Affiliations:** 1 McGill University Health Center Research Institute, Department of Infectious Diseases, McGill University, Montreal, Quebec, Canada; 2 Segal Cancer Centre, Lady Davis Institute for Medical Research, SMBD Jewish General Hospital, McGill University, Montreal, Quebec, Canada; 3 Department of Microbiology & Immunology, Dalhousie University, Halifax, Nova Scotia; Institut Pasteur, France

## Abstract

Vitamin A can significantly decrease measles-associated morbidity and mortality. Vitamin A can inhibit the replication of measles virus (MeV) *in vitro* through an RARα- and type I interferon (IFN)-dependent mechanism. Retinoid-induced gene I (RIG-I) expression is induced by retinoids, activated by MeV RNA and is important for IFN signaling. We hypothesized that RIG-I is central to retinoid-mediated inhibition of MeV *in vitro*. We demonstrate that RIG-I expression is increased in cells treated with retinoids and infected with MeV. The central role of RIG-I in the retinoid-anti-MeV effect was demonstrated in the Huh-7/7.5 model; the latter cells having non-functional RIG-I. RAR-dependent retinoid signaling was required for the induction of RIG-I by retinoids and MeV. Retinoid signaling was also found to act in combination with IFN to induce high levels of RIG-I expression. RIG-I promoter activation required both retinoids and MeV, as indicated by markers of active chromatin. IRF-1 is known to be regulated by retinoids and MeV, but we found recruitment of IRF-1 to the RIG-I promoter by retinoids alone. Using luciferase expression constructs, we further demonstrated that the IRF-1 response element of RIG-I was required for RIG-I activation by retinoids or IFN. These results reveal that retinoid treatment and MeV infection induces significant RIG-I. RIG-I is required for the retinoid-MeV antiviral response. The induction is dependent on IFN, retinoids and IRF-1.

## Introduction

In 2007 measles was responsible for 197 000 deaths, the lowest rate ever reported [1]. Although improved vaccine coverage is the primary factor driving this change, increased survival of those infected through the use of high dose vitamin A (retinol) has likely made an important contribution. Morbidity and mortality due to measles can be significantly reduced with two doses of retinol (200,000 IU; water-based formulation) [2], [3], [4]. Since the mid-1990s, the WHO and UNICEF have recommended vitamin A treatment for acute measles in regions of the developing world with high mortality rates [5].

Vitamin A, its synthetic derivatives and metabolites are collectively referred to as retinoids [4]. Retinoids are required for a wide-range of crucial biological processes including regulation of embryonic development, maintenance of the integrity of epithelial cell surfaces, vision and immunity [6]. The active retinol metabolite, all trans retinoic acid (ATRA) is responsible for mediating many of the important functions of retinoids. ATRA is the natural ligand for the retinoic acid receptors (RARs), which form heterodimers with the retinoid X receptors (RXRs) within the nucleus [7]. RAR-RXR heterodimers bind to retinoid acid response elements (RAREs) on the promoters of target genes to activate transcription of these genes when bound by ligand [4].

Recent studies from our group have reported that ATRA can inhibit measles virus (MeV) replication *in vitro* through a retinoid nuclear receptor-dependent pathway [8]. Our studies further showed that interferon (IFN) is necessary for this anti-viral effect, and that initially uninfected bystander cells are protected from subsequent viral infection by up regulating the expression of IFN-stimulated genes (ISGs) [9]. Anti-MeV effects of retinoids have been demonstrated in a number of primary human cells and cell lines of diverse tissue origin [8] including myelomonocytic U937 cells that have been an important model for these molecular studies.

Retinoids are implicated in regulating the expression of a number of ISGs, including retinoid-induced gene I (RIG-I) and IFN regulatory factor 1 (IRF-1) [10], [11], [12], [13], [14], [15], [16], [17], [18], [19]. RIG-I is a pattern recognition receptor that can detect single-stranded RNA [20], [21], [22]. RIG-I is expressed at a basal level in many cell types. It can initiate the production of type I IFN and is itself an ISG [23]. IFN has been reported to induce RIG-I expression by causing the IRF-1 transcription factor to bind to the RIG-I promoter [24].

The RIG-I ligand has been shown to be 5′-triphosphorylated, short single-stranded RNA [25], although other ligands have been identified (reviewed in [26]). RIG-I has been shown to recognize a variety of RNA viruses, including MeV [22]. To investigate the requirement of RIG-I signaling in response to retinoids and MeV, we used the Huh-7 cell line, which is derived from a human hepatocellular carcinoma used extensively in hepatitis C virus (HCV) research [27], [28]. Of particular interest for our studies, an Huh-7 subclone (Huh-7.5) that is permissive for HCV RNA replication [28] has a transition point mutation of a C to T at nucleotide 164 in the CARD domain of RIG-I rendering the protein non-functional RIG-I [29], [30].

RIG-I was originally identified as a retinoid-responsive gene by treating NB4 cells with 1 µM of ATRA for 48 hours [10]. The NB4 cell line is derived from acute promyelocytic leukemia (APL) with a t(15∶17) reciprocal translocation [31]. This translocation fuses the PML gene with the retinoic acid receptor alpha (RARα) generating a PML-RARα chimera [32], [33], [34], [35], [36]. The fusion protein retains functional domains of RARα and has been shown to be a ligand-dependent transcriptional activator of RAREs [33], [34], [35]. A subclone of NB4 cells, NB4-MR4 (R4 cells), are retinoic acid resistant due to a point mutation in the ligand-binding domain of the fusion PML-RARα [37]. Mutant PML-RARα proteins do not bind ligand but retain their ability to bind to RAREs and block the transcription of retinoic acid responsive genes in a dominant-negative fashion [37]. This model facilitated investigation of the role of retinoid signaling in the induction of RIG-I and the retinoid-induced anti-MeV state.

We hypothesize that RIG-I is essential for the retinoid mediated anti-MeV response and that the inhibition of MeV requires both RAR-RXR activity and an IFN signal [8], [9].

## Results

### RIG-I expression is regulated by the combination of MeV infection and ATRA treatment

**Figure 1:**
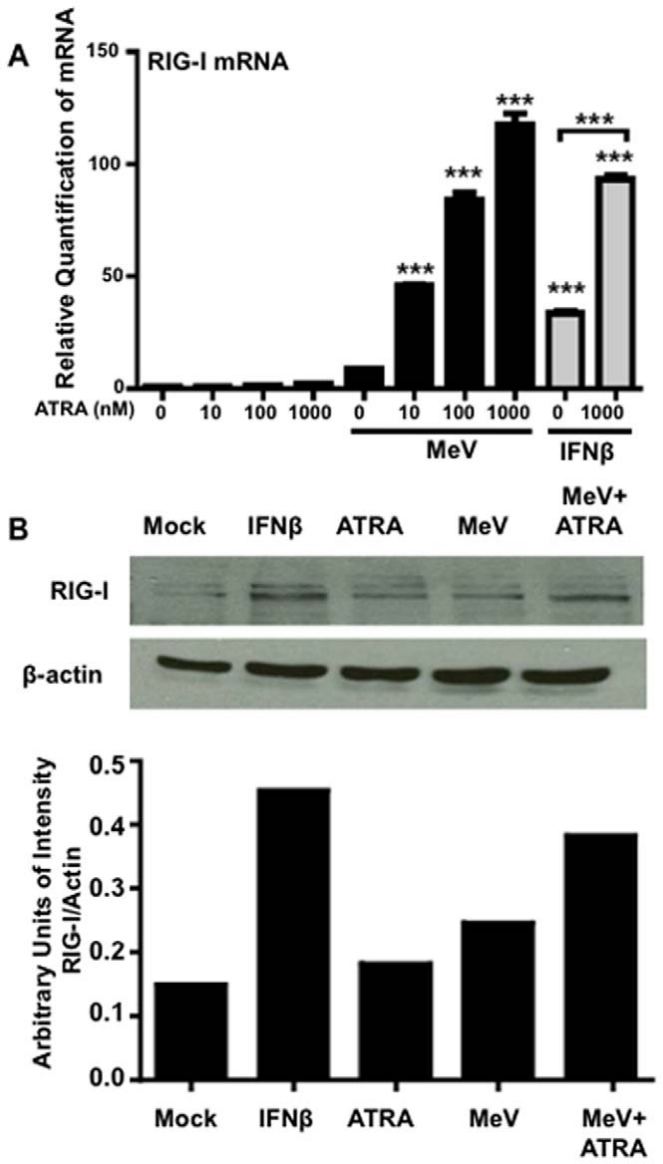
RIG-I expression is regulated by MeV and ATRA. (A) U937 cells were infected with MeV at an MOI of 0.1 and treated with increasing doses of ATRA or DMSO for 24 hours. Some samples were also treated with 1000 U/mL IFNβ for 24 hours, with or without ATRA. RNA was extracted and analyzed for RIG-I expression by qPCR. Data presented are representative of three experiments performed in triplicate (N = 3). (B) U937 cells were infected with MeV at an MOI of 0.1 and treated with ATRA or DMSO (1 µM) for 48 hours, or with 2000 U/mL IFNβ as a positive control. Samples were analyzed by western blot for RIG-I and β-actin expression and quantified. ***p<0.001.

We have previously shown that MeV can be inhibited in a number of cells lines including U937 cells and PBMCs [8]. To determine the involvement of RIG-I in the retinoid-mediated inhibition of MeV, the regulation of RIG-I expression during MeV infection with and without ATRA treatment was investigated in U937 cells. These cells are neoplastic and histiocytic progenitors of monocytes that have been extensively used in immunological studies [38]. They can be infected with MeV and are partially responsive to pharmacological doses of retinoids [8]. RIG-I mRNA and protein are expressed at very low levels in untreated U937 cells. MeV infection alone resulted in a small increase in RIG-I mRNA, while ATRA treatment alone had no discernible effect on RIG-I expression in this cell line. Importantly, U937 cells infected with MeV and treated with increasing doses of ATRA showed a dose response in RIG-I expression at the mRNA level (Figure 1A) and increased expression at the protein level (Figure 1B) over the induced over-expression of RIG-I by the artificial treatment with exogenous IFNβ. The IFNβ (positive control) could induce RIG-I expression as expected (Figure 1A). The combination of ATRA and IFNβ treatment resulted in higher levels of RIG-I expression than IFNβ alone (Figure 1A). Additionally, in our system we observe the up-regulation of a number of ISGs including IRF-7 [9] and MDA-5 (data not shown). RIG-I and MDA-5 have both been implicated in the induction of IFN in response to MeV [39]. The importance of RIG-I in this antiviral response due to its regulation by retinoids. To date, there has been no evidence that MDA-5 is a retinoid responsive gene.

### RIG-I necessary for anti-viral effect of retinoids

RIG-I is an interferon-stimulated gene (ISG) involved in the positive feedback loop that contributes to increased expression of type I IFN and the induction of an antiviral state. Previous findings from our group have demonstrated that other ISGs, such as IRF-7, are up-regulated in response to ATRA treatment during MeV infection [9]. RIG-I is clearly up-regulated by the combination of ATRA and MeV in our system (Figure 1A, 1B), therefore its requirement in mediating the anti-viral effect was further investigated. We used the Huh-7/7.5 cell culture model of RIG-I functional loss rather than RNA interference (RNAi) because we observed that both control and RIG-I specific siRNA (as well as siRNA specific to other genes in the RIG-I pathway) were sufficient to induce the expression of RIG-I and other interferon stimulated genes using the latter approaches (data not shown, also demonstrated in [25], [40]).

**Figure 2:**
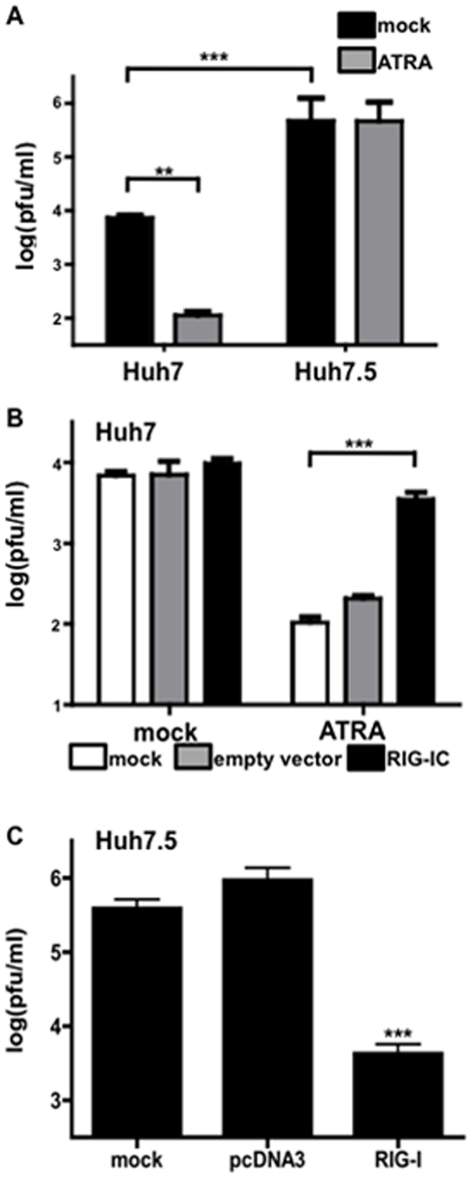
RIG-I necessary for inhibition of MeV by ATRA. (A) Huh7 and Huh7.5 cells were infected MeV at an MOI of 0.01 and treated with 1 µM ATRA or DMSO. Whole cell lysates were harvested after 48 hours and viral titers were measured by plaque assay. (B) Huh7 cells were infected with MeV at an MOI of 0.01 and treated with 1 µM ATRA or DMSO and transfected with the control plasmid or RIG-I dominant negative (RIG-IC) expression construct. Whole cell lysates were harvested after 48 hours and viral titers were measured by plaque assay. (C) Huh7.5 cells were transfected with the control plasmid or RIG-I expression construct and infected with MeV at an MOI of 0.01. Whole cell lysates were harvested after 48hr and viral titers were measured by plaque assay. Data represent two experiments performed in triplicate (N = 2). **p<0.01, ***p<0.001.

As expected in the Huh-7/7.5 system, Huh-7.5 cells with non-functional RIG-I permitted higher levels of viral replication compared to the RIG-I functional Huh-7 cells (Figure 2A). Levels of RIG-I mRNA and protein are comparable in both cell lines (data not shown). ATRA treatment significantly inhibited MeV production in Huh-7 cells but had no effect in Huh-7.5 cells (Figure 2A), suggesting that RIG-I has a central role in the inhibitory effect of retinoids.

To confirm the role of RIG-I in mediating the anti-MeV effect of ATRA, a RIG-I dominant-negative mutant containing only the helicase domain of RIG-I (RIG-IC) [23] was transfected into the ATRA-responsive Huh-7 cells (Figure 2B). RIG-IC has been previously shown to inhibit Influenza A virus-induced IFN expression [41]. When Huh-7 cells were infected with MeV and treated with ATRA, then transfected with the dominant negative mutant, there was no reduction of MeV replication (Figure 2B). Blocking RIG-I with RIG-IC in the absence of ATRA treatment had no significant impact on MeV production (Figure 2B).

Previous studies have demonstrated that RIG-I complementation in Huh7.5 cells restores the IRF3 pathway rendering the cells responsive to Sendai virus infection [30]. This demonstrates that the non-functional RIG-I encoded in the Huh7.5 cells can be complemented by exogenous expression of the protein. When wildtype RIG-I is expressed in Huh7.5 cells MeV is inhibited (Figure 2C).

When RIG-I was blocked in Huh7 cells, the retinoid-MeV anti-viral effect was lost. Additionally, over-expression of RIG-I can restore the ability of Huh7.5 cells to inhibit MeV. These data confirms the requirement of RIG-I for the induction of the retinoid-MeV anti-viral response.

### IFN production up-regulates RIG-I expression in bystander cells

In different cell culture models MeV can inhibit IFN signaling to varying degrees and is strain dependent [42]. The tyrosine residue at the shared position 110 of the V and P proteins is necessary for MeV to block Stat1 phosphorylation [42], [43]. Sequencing of the Chicago stain of MeV used in these experiments demonstrates a tyrosine a position 110 suggesting that the V protein is functional [9]. In our previous studies we have demonstrated increased expression of IFNα1 mRNA by qPCR and IFNα1 protein expression in the supernatant of cells treated with ATRA+MeV [9]. We have also demonstrated that IFN signaling is important for the induction of ISGs in the context of ATRA treatment and MeV infection [9]. Type I IFN is a key modulator of the innate immune response to viral infection and, RIG-I is an ISG [22]. We next sought to determine the role of IFN signaling in mediating the anti-MeV actions of RIG-I.

**Figure 3:**
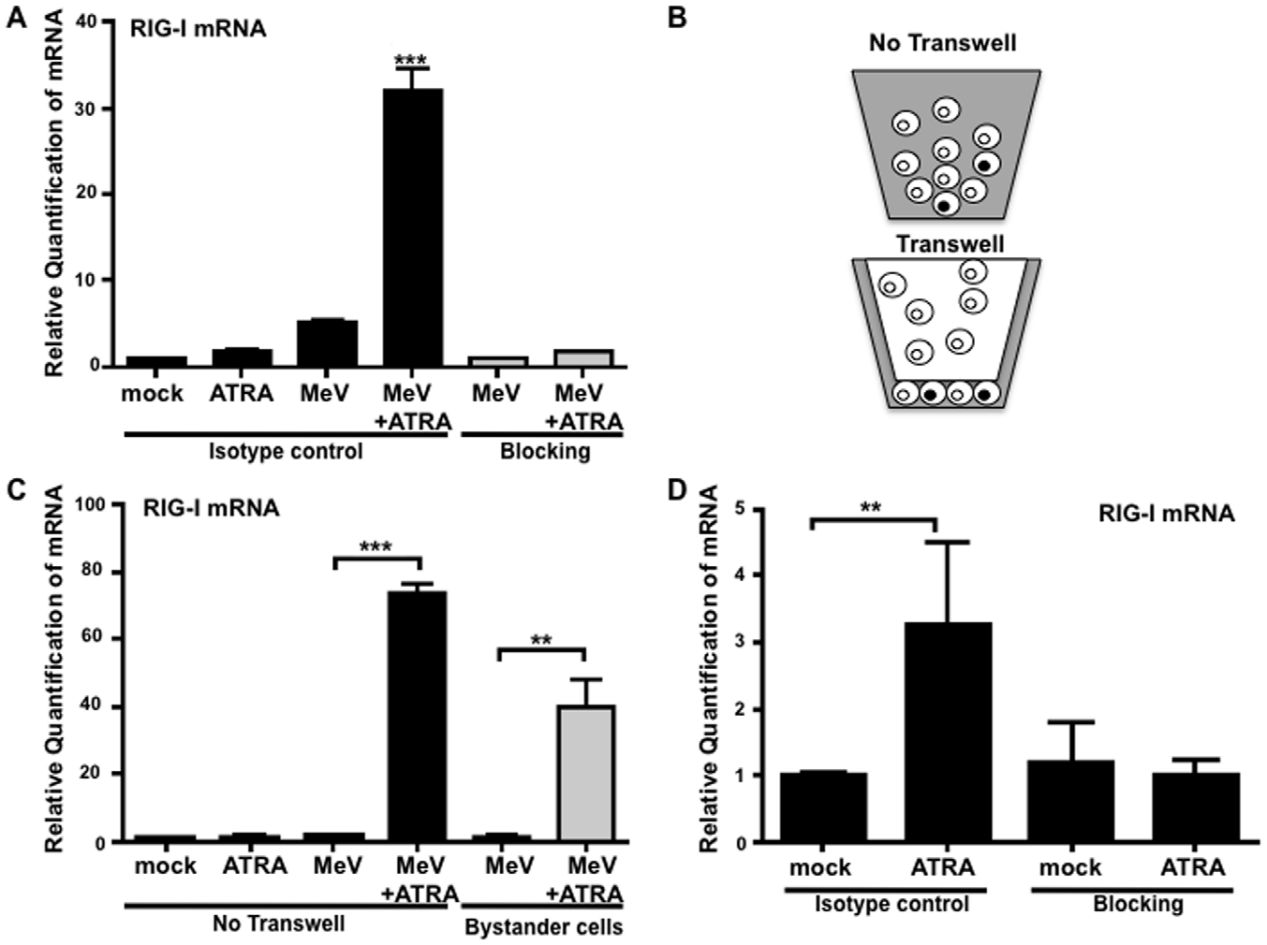
MeV with ATRA induces the soluble factor IFN to elicit the expression of RIG-I. (A) Cells were infected with MeV at an MOI of 0.1 in the presence of 1 µM ATRA or DMSO, and either IFNαβ-receptor blocking antibodies or isotype control. RNA was extracted at 24 h and RIG-I expression was measured by qPCR. Data presented are representative of 2 experiments performed in triplicate (N = 2). (B) Transwell membrane inserts with 0.02 µm pores were used to separate the infected cells from the uninfected, bystander cells in the inner chamber [9]. (C) Cells from transwell-free control wells and the inner chamber bystander cells were harvested after 48 hours and RIG-I mRNA was measured by qPCR. Data presented are representative of three experiments performed in triplicate (N = 3). (D) Supernatants from the control wells and the inner chambers of the transwells were used to treat fresh U937 cells with either IFNαβ-receptor blocking antibody or isotype control antibody. Following 24 hours of incubation, RIG-I expression was assessed by qPCR. Data presented are representative of three experiments performed in triplicate (N = 3). **p<0.01, ***p<0.001.

In this study, we have confirmed our previous findings demonstrating the presence of IFNα1 and IFNβ in the supernatant of the ATRA+MeV cells (data not shown). The substantial induction of RIG-I mRNA by ATRA+MeV was found to be abrogated when U937 cultures were treated with IFN α/β receptor blocking antibodies (Figure 3A), in agreement with our previous findings [9]. A transwell system (Figure 3B) was used to determine if up-regulation of RIG-I could be induced in bystander cells, not in contact with the virus, as previously described for IRF-7 [9]. As predicted, there was a robust up-regulation of RIG-I mRNA in bystander cells exposed to ATRA-treated, MeV-infected cells in the transwell system (Figure 3C).

The effect of conditioned media from the inner (uninfected) transwell chamber on RIG-I expression in fresh U937 cells was analyzed by qPCR. Media conditioned by exposure to ATRA-treated, MeV-infected cells in the transwell was sufficient to induce RIG-I expression, whereas media conditioned by exposure to mock-treated, MeV-infected cells failed to do so (Figure 3C). Importantly, the up-regulation of RIG-I by inner-chamber media exposed to both ATRA-treated and MeV-infected cells could be blocked by antibodies targeting type I IFN α/β receptor (Figure 3D). These data show that IFN is critical for the induction of both RIG-I and the retinoid-mediated anti-viral state.

### RARα signaling is required for the induction of RIG-I

We have shown that RIG-I is required for induction of the ATRA-mediated anti-MeV response. RIG-I is both a retinoid-responsive gene [10] and a cytoplasmic pattern-recognition receptor that recognizes MeV [22]. To define the role of retinoid signaling in the induction of RIG-I and the retinoid-induced anti-MeV state, we used the NB4/R4 cell model (retinoid signaling responsive versus resistant respectively) [37]. Our group has recently used this model to demonstrate that RARα signaling is important for the inhibitory effect of retinoids against MeV [8]. Additionally, we have used this model to demonstrate the inhibition of MeV by retinoids in the NB4 cells, but not in the R4 cells [8].

**Figure 4:**
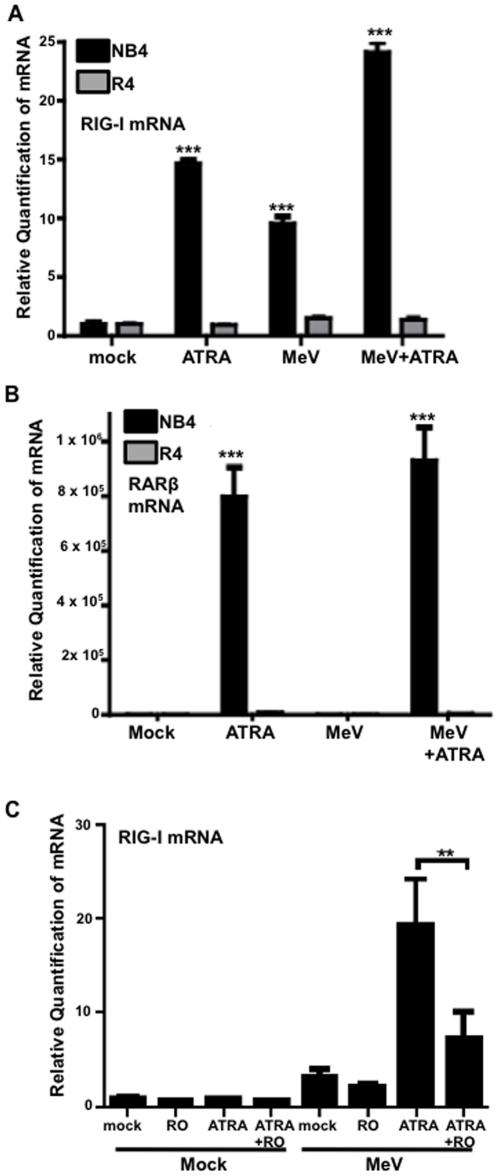
RIG-I expression required RAR signaling. (A) NB4 and R4 cells were infected with MeV at an MOI of 0.01, treated with 1 µM ATRA or DMSO and/or treated with 1000 U/mL IFNβ. After 48 hours, samples were harvested and analyzed for RIG-I expression and (B) RARβ expression by qPCR. Data presented are representative of two experiments performed in triplicate (N = 2). (C) U937 cells were infected at an MOI of 0.1 for 24 hours in the presence of 10 nM ATRA and/or 1000 nM of the RARα-selective antagonist RO 41-5253 (RO). Samples were analyzed for RIG-I expression by qPCR. Data presented are representative of three experiments performed in triplicate (N = 3). **p<0.01, ***p<0.001.

In NB4 cells, ATRA treatment alone had the ability to induce modest levels of RIG-I mRNA as measured by qPCR (Figure 4A) and western blot (data not shown). MeV infection of these cells by itself also induced moderate increases in RIG-I expression (Figure 4A, data not shown). Similar to the U937 cell model (Figure 1), the combination of ATRA treatment and MeV infection yielded a significant level of RIG-I mRNA (Figure 4A, data not shown). In R4 cells, resistance to retinoid signaling was first confirmed by demonstrating the inability of ATRA to induce the expression of RARβ, a well documented retinoid responsive gene (Figure 4B). In these cells, neither ATRA treatment alone, nor MeV infection alone, nor the combination could induce the expression of RIG-I mRNA (Figure 4A) or protein (data not shown). It is important to note that the antiviral effect of ATRA on MeV in NB4 cells is not due to retinoid induced cell differentiation as previously demonstrated by CD11b expression [8]. Increased apoptosis of NB4 cells is also not the cause of the antiviral effect as demonstrated in our previous work [8]. These observations demonstrate a requirement of RAR signaling for the induction of RIG-I.

To confirm the importance of RARα signaling in the expression of RIG-I, in our original model, we used the RARα antagonist RO in U937 cells [44]. RO treatment successfully blocked the induction of RIG-I mRNA observed with the combination of ATRA treatment plus MeV infection (Figure 4C). This finding lends further support to the important role of RARα signaling in the regulation of RIG-I expression.

### Retinoic Acid nuclear receptors occupy the RIG-I promoter

Retinoid signaling is well documented to be important for many crucial functions in the cell including and, as our data show, the regulation of RIG-I expression. Therefore, we explored the recruitment of relevant protein complexes and chromosomal changes occurring at the RIG-I promoter.

**Figure 5:**
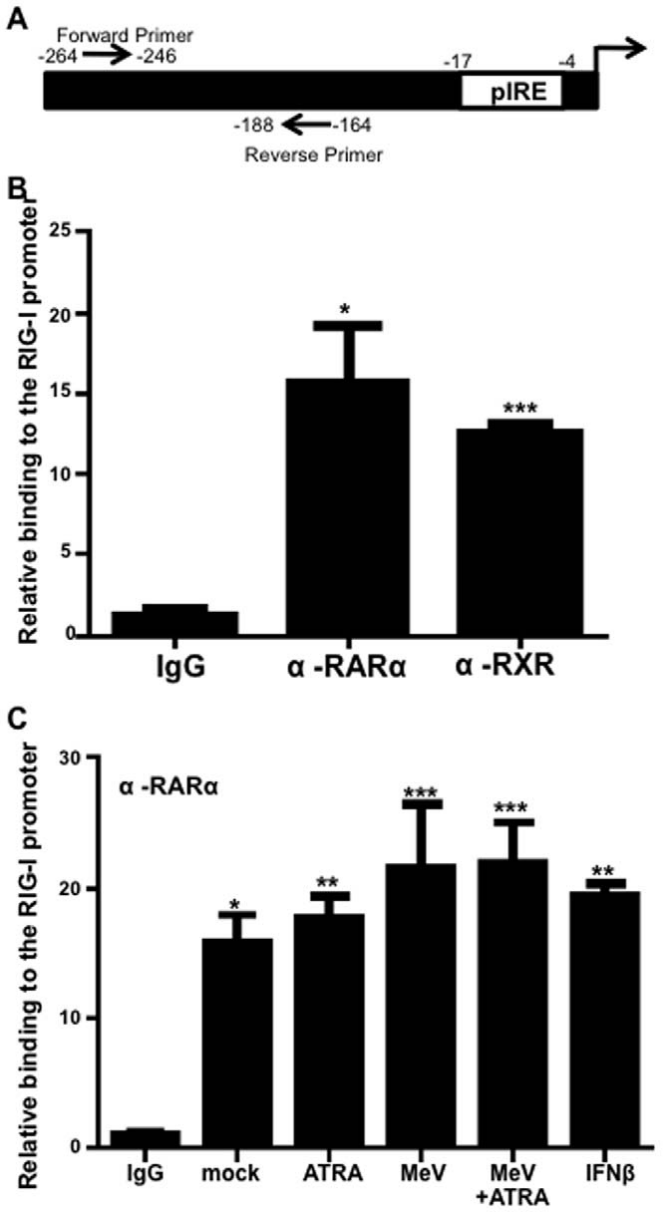
Retinoic acid nuclear receptors bind to the RIG-I promoter. (A) Diagram of the RIG-I promoter showing the known IRF1 binding site. Arrows represent the site of primers used in ChIP experiments. (B) RARα and RXR were immunoprecipitated from cells treated with 1 µM ATRA or DMSO (N = 2) (C) U937 cells were infected with MeV at an MOI of 0.1 and/or treated with 1 µM ATRA or DMSO for 24 hours. 1000 U/ml of IFNβ was used as a positive control. These samples were then immunoprecipitated RARα primary antibodies. The pulled-down DNA was analyzed by qPCR using primers specific for the RIG-I promoter as described in the materials and methods. Data presented are representative of three experiments performed in triplicate (N = 3). *p<0.05, **p<0.01, ***p<0.001.

Fourteen putative retinoic acid response elements (RARE) were identified within 10000 bp of the RIG-I start site using Genomatix MatInspector software. Predicted RAREs were confirmed using the consensus sequences previously described [45]. For a DR5/DR2, the consensus sequence is: not C, G, G/T, not A, G/C, A, 2 or 5 nucleotides, A/G, G, G/T, G/T, C/A, A [45]. Using Chromatin Immunoprecipitation (ChIP) assays in our U937 model (Figure 5A), we found that both RARα and RXR bind to the RIG-I promoter (Figure 5B). Retinoid nuclear receptor binding to an RARE is not dependent on ligand binding [46]. RARα (Figure 5C) and RXR (data not shown) binding to the RIG-I promoter was not affected by treatment with either ATRA or IFNβ, or by MeV infection.

### Activation of RIG-I promoter only upon combination of MeV and ATRA treatment

**Figure 6:**
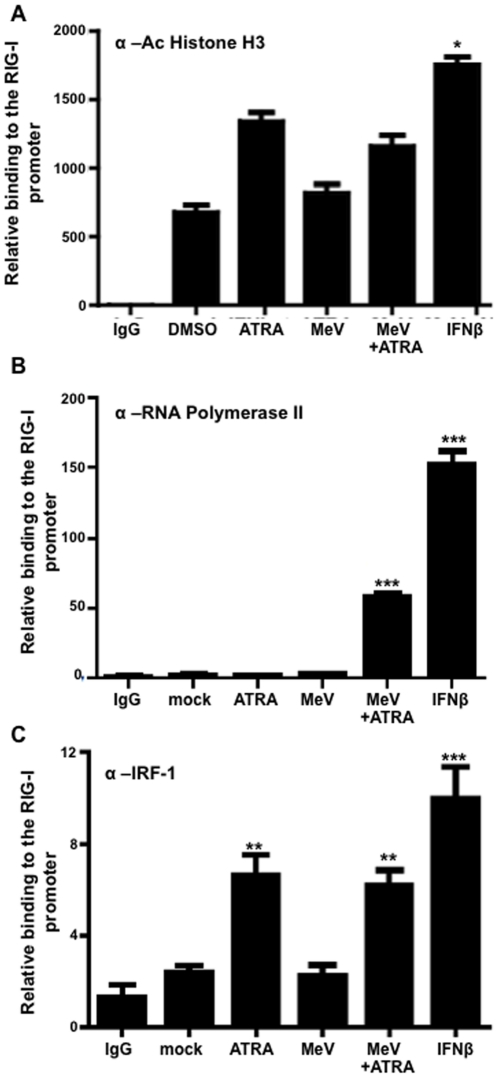
Activation of RIG-I promoter only upon combination of MeV infection and retinoid treatment. U937 cells were infected with MeV at an MOI of 0.1 and/or treated with 1 µM ATRA or DMSO for 24 hours. 1000 U/ml of IFNβ was used as a positive control. These samples were then immunoprecipitated the following primary antibodies (A) Acetylate Histone H3 (B) Pol II (C) IRF-1. The pulled-down DNA was analyzed by qPCR using primers specific for the RIG-I promoter as described in the materials and methods. Data presented are representative of experiments performed in triplicate between two and three times (N = 2–3). *p<0.05, **p<0.01, ***p<0.001.

To better define the effect of retinoids ± MeV on the transcriptional regulation of RIG-I, chromatin remodeling and the recruitment of proteins associated with transcriptional activation on the RIG-I promoter were investigated in our U937 model. As an indication of chromatin remodeling, acetylation of histone H3 (Figure 6A) and histone H4 (data not shown) was increased following combined ATRA treatment and MeV infection compared to either manipulation alone and significantly increased over control. Further, RNA Polymerase II (Pol II) was strongly recruited to the RIG-I promoter only by IFNβ treatment (positive control) or by the combination of ATRA treatment plus MeV infection (Figure 6B).

### IRF-1 expression is regulated by Measles Virus and ATRA

Regulation of the RIG-I promoter is not limited to retinoic acid nuclear receptors and traditional transcription factors. Previous reports have shown that IRF-1 binds the RIG-I promoter and is important in regulating the IFNβ-mediated up-regulation of RIG-I expression [24]. We therefore used ChIP assays in our U937 model to determine the impact of ATRA ± MeV on IRF-1 binding to the RIG-I promoter. In our hands, IRF-1 binding to the RIG-I promoter was readily detected following IFNβ treatment, as has been previously described (Figure 6C) [24]. Interestingly, ATRA alone and the combination of MeV and ATRA also induced IRF-1 binding to the RIG-I promoter in U937 cells (Figure 6C). Therefore, ATRA alone appears to be sufficient to recruit IRF-1 to the RIG-I promoter, but is insufficient for the induction of RIG-I expression. Only IFNβ treatment and the ATRA+MeV combination were successful at eliciting the expression of both RIG-I mRNA and protein (Figure 1A and 1B).

In addition to regulation of the RIG-I promoter, several groups have demonstrated that IRF-1 is induced and activated by ATRA treatment [11], [12], [13], [14], [15], [16], [17], [18], [19]. It was therefore of interest to determine what effect, if any, MeV infection and combined ATRA treatment plus MeV infection would have on the regulation of IRF-1.

**Figure 7:**
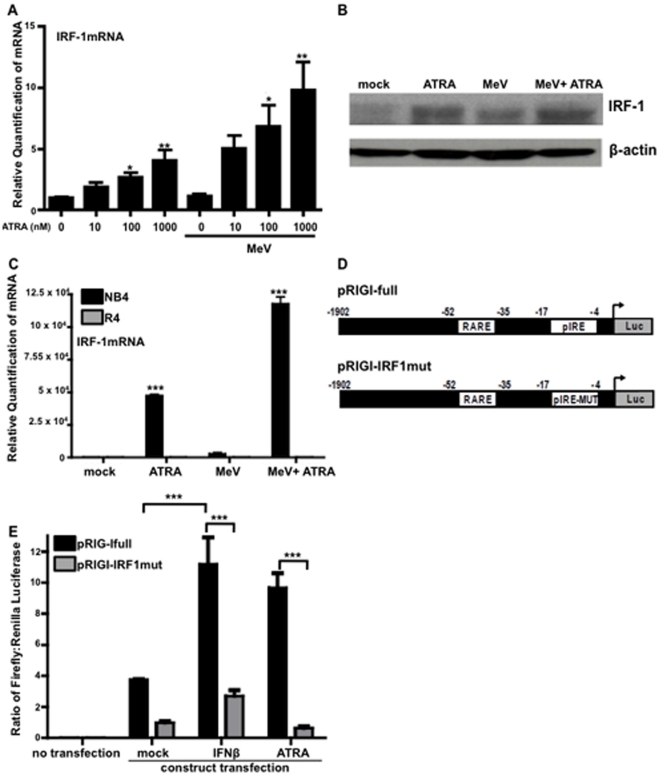
IRF-1 is regulated by MeV infection and retinoid treatment contributing to the RIG-I antiviral response. (A) U937 cells were infected with MeV at an MOI of 0.1 and treated with increasing doses of ATRA or DMSO for 24 hours. RNA was extracted and analyzed for IRF1 expression by qPCR. Data presented are representative of three experiments performed in triplicate (N = 3). (B) U937 cells were infected with MeV at an MOI of 0.1 and/or treated with 1 µM ATRA or DMSO for 24 hours. Cells were also treated with 1000 U/ml of IFNβ as a positive control. Samples were analyzed by western blot for IRF-1 and β-actin expression. (C) NB4 and R4 cells were infected with MeV at an MOI of 0.01, treated with 1 µM ATRA or DMSO and/or treated with 1000 U/mL IFNβ. After 48 hours, samples were harvested and analyzed for IRF-1 expression by qPCR. Data presented are representative of three experiments performed in triplicate (N = 3). (D) A schematic of the RIG-I promoter constructs including the full length construct pRIGI-full and the IRF-binding mutant pRIGI-IRFmut. (E) HeLa cells were transfected with 3 µg of pRIGI-full or pRIGI-IRFmut construct and 0.5 µg of Renilla control. The cells were then treated with 1 µM ATRA or 1000 U/ml of IFNβ for an additional 24 hours. Samples were then analyzed for dual-luciferase expression. *p<0.05, **p<0.01, ***p<0.001.

In our U937 model, we observed that both IRF-1 mRNA and protein could be induced by ATRA alone, as well as with the combination of ATRA treatment and MeV infection (Figures 7A and 7B). MeV infection alone did not result in detectable changes in IRF-1 mRNA, but induced modest amounts of IRF-1 protein (Figures 7A and 7B).

Further studies in our NB4/R4 model revealed this induction of IRF-1 mRNA by ATRA alone or in combination with MeV infection to be RARα dependent. When NB4 cells are treated with ATRA and infected with MeV or treated with ATRA alone there is a remarkable induction of IRF-1 mRNA (Figure 7C). In R4 cells, neither ATRA treatment nor the combination of ATRA and MeV was able to induce the expression of IRF-1 mRNA (Figure 7C).

### IRF-1 binding to the RIG-I promoter is required for RIG-I induction

To investigate the requirement of IRF-1 binding the RIG-I promoter to induce an antiviral state, we employed synthetic luciferase constructs targeting the region from −1902 bp to −1 bp of the start site of the RIG-I promoter [23], [24] (Figure 7D). The native construct (pRIGI-full) includes both the known IRF-1 binding site and a predicted RARE (Figure 7D). A second construct, pRIGI-IRFmut, that contains a mutation in the IRF-1 binding site was used to determine the requirement of IRF-1 binding in the ATRA-mediated transcription of the RIG-I promoter. Promoter construct experiments were conducted in HeLa cells for ease of transfection. HeLa cells do not demonstrate the retinoid-antiviral phenotype due to atypical IFN signaling (data not shown).

When HeLa cells were transfected with pRIGI-full, we found that IFNβ treatment could induce expression from the RIG-I promoter, as predicted (Figure 7E). ATRA treatment alone also strongly induced expression from this promoter construct (Figure 7E). Although we do not see the expression of RIG-I in U937 cells (Figure 1A), we have observed the expression of RIG-I following ATRA treatment alone in NB4 cells (Figure 4A) and in other cell lines (data not shown). However, neither IFNβ nor ATRA could induce expression from the pRIGI-IRFmut construct, demonstrating a requirement of activation of the IRF-1 response element in the RIG-I promoter for the expression of RIG-I (Figure 7E).

## Discussion

Retinol (Vitamin A) is known to have significant clinical benefit in natural MeV infection, and we have shown that retinoids can have powerful anti-MeV effects *in vitro* [2], [3], [8]. Specifically, we found that intact retinoid nuclear receptor signaling, as well as functional type I IFN signaling, are necessary for this inhibitory effect [8], [9]. These studies also revealed that the antiviral impact of retinoids in our *in vitro* models was largely realized in cells not yet productively infected by MeV (so-called ‘bystander cells’ [9]) due to their exposure to IFN and the resultant high level ISG expression [9]. In the current work, we sought to explain the mechanism that leads to the large burst of IFN that protects these bystander cells from infection. Because RIG-I is an important component of IFN signaling, and known to be regulated by ATRA [10], [23], we hypothesized that this molecule could act as a bridge between retinoid and IFN signaling to mediate the inhibition of MeV.

The RIG-I data presented herein, using a range of *in vitro* models, extends our previous observations showing the induction of IRF-7 by MeV and ATRA. In both studies, type I IFN and RARα signaling are required [9]. We have now extended these findings to show that the combination of ATRA treatment and MeV infection effectively up-regulates RIG-I at the mRNA and protein levels in both U937 and NB4 cells (Figure 1A, 1B, 4A, 4C), and that up-regulation of RIG-I expression is required to create the ATRA-MeV anti-viral state (Figure 2A).

Our previous work had also demonstrated that the antiviral state created by ATRA treatment during MeV infection occurred in uninfected bystander cells and was dependent on type I IFN [9]. In the current study, we show that RIG-I is up-regulated in these uninfected cells (Figure 3C), and that type I IFN secreted into the media is responsible for inducing the antiviral state (Figure 3A and 3D). When the IFN α/β receptor is blocked with monoclonal antibodies, the induction of RIG-I is lost (Figure 3A), consistent with our finding that IFN production is required for the antiviral state induced by combined ATRA treatment plus MeV infection.

To investigate the role of retinoid signaling in the induction of RIG-I and its connection to the retinoid-induced MeV anti-viral state, we used the NB4/R4 cell model (which differ in functional versus non-functional nuclear retinoid receptor signaling [37]). In the retinoid-responsive NB4 cells, combined ATRA treatment and MeV infection resulted in a strong induction of RIG-I mRNA and protein (Figure 4A, data not shown). In contrast, blunted RAR signaling in the retinoid-resistant R4 cells prevented the combination of ATRA treatment plus MeV infection from inducing RIG-I mRNA expression (Figure 4A, Figure data not shown). Functional retinoid signaling via RAR is therefore required for the robust induction of RIG-I by combined ATRA treatment plus MeV infection.

A number of putative RARE were identified on the RIG-I promoter, many of had high sequence homology with the consensus sequences predicted by Balmer and Blomhoff [45]. The retinoid nuclear receptor heterodimer, RAR+RXR, was shown to bind to the RIG-I promoter (Figure 5B). This binding was not influenced by ATRA treatment or MeV infection (Figure 5C and data not shown). These data suggest that regulation of the RIG-I promoter may be influenced by the presence of one or more RARE and confirm that RIG-I is a retinoid responsive gene. Despite binding of the nuclear receptors to the RIG-I promoter, induction of RIG-I mRNA by ATRA alone was not observed in U937 cells (Figure 1A) and is only minimally observed in NB4 cells (Figure 4A). Thus, additional activating events at the RIG-I promoter may be needed.

Using ChIP, markers of chromatin remodeling, acetylation of histone 3 (Figure 6A) and acetylation of histone 4 (data not shown) were seen to increase during ATRA treatment with MeV infection. These findings indicate that the RIG-I promoter is rendered conducive to the initiation of transcription through exposure to combined ATRA treatment plus MeV infection. This supposition is strongly supported by the recruitment of Pol II to the RIG-I promoter only under the conditions of ATRA treatment plus MeV infection (Figure 6B). This regulation of the promoter correlates with Figure 1A, which shows the induction of RIG-I mRNA only during combined ATRA treatment and MeV infection.

The name, Retinoid Induced Gene-I, implies that RIG-I is indeed retinoid inducible. However, our data reveal only moderate retinoid responsiveness in some cell lines, such as NB4 (Figure 4A), but no induction whatsoever in other cell lines such as U937 in response to ATRA (Figure 1A). In the initial microarray screen performed by Liu et al. that identified the RIG genes, including RIG-I, cyclohexamide was observed to prevent the induction of RIG-I following ATRA treatment [10]. This suggested that RIG-I is not directly inducible by ATRA, despite the presence of putative RARE sequences and readily demonstrated binding of the nuclear receptors to the RIG-I promoter (Figure 5A–C).

RIG-I was recently shown to be up-regulated by IFN through the transcriptional regulation of its promoter by IRF-1 [24]. IRF-1 was the first transcription factor shown to regulate the IFNβ promoter and is also an ISG [47], [48]. Although post-translational modifications have been implicated in controlling its ability to induce transcription [49], [50], [51], [52], IRF-1 itself is regulated primarily at the level of transcription [53]. Several groups have found that ATRA can rapidly induce IRF-1 expression [11], [12], [13], [14], [15], [16], [17], [18], [19]. Recently, ATRA has been shown not only to induce IRF-1, but also increase to its nuclear localization and binding to specific promoters [18]. This effect appears to occur through an RARα-dependent pathway [18].

In the present study, we confirmed that IRF-1 is induced by ATRA alone in U937 cells and that ATRA treatment causes IRF-1 binding to the RIG-I promoter (Figure 7A–B, Figure 6C). The level of IRF-1 expression was further increased when ATRA-treated cells were infected with MeV (Figure 7A). Also, the induction of IRF-1 appears to be RAR-dependent, as shown by the lack of expression in R4 cells deficient in retinoid signaling (Figure 7C). IRF-1 binding to the RIG-I promoter in ATRA-treated cells was not influenced by MeV infection (Figure 6C). However, indicators of transcriptional activation, such as recruitment of Pol II, as well as the presence of RIG-I mRNA, were only observed following the combination of ATRA treatment and MeV infection in U937 cells (Figure 6B and 1A). In our hands, IFNβ treatment was sufficient to induce IRF-1 expression, IRF-1 binding to the RIG-I promoter and RIG-I expression. These data support the findings of Su et al. and suggest that IRF-1 is primarily responsible for the IFN-induced expression of RIG-I (Figures 7A-B and data not shown).

Since both RIG-I and IRF-1 are induced by combined ATRA treatment and MeV infection, but RIG-I appears to be essential for the antiviral response, we examined the requirement of IRF-1 for the induction of RIG-I using a RIG-I promoter construct containing both a hypothetical RARE and the IRF-1 response element (IRE) [24] (Figure 7D). The RIG-I promoter initiated expression following both IFN and ATRA treatment (Figure 7E) but neither treatment could initiate promoter expression when the IRE was mutated (Figure 7E). These findings show that the RIG-I promoter is regulated by ATRA in an IRF-1-dependent manner. Lou et al. have recently reported that the induction of retinoid-induced gene G (RIG-G) is also mediated primarily through IRF-1 [54].

Our studies to date lead us to propose a model in which transcriptional up-regulation of RIG-I acts as a critical step in the inhibition of MeV by ATRA. In particular, we have demonstrated that when a cell population is infected with MeV at a low multiplicity of infection (MOI) and treated with ATRA, the initially uninfected cells are protected from productive infection by exposure to large amounts of IFN and the up-regulation of ISG expression as a bystander effect [9]. We hypothesize that initially uninfected cells exposed to retinoids and media conditioned by infected cells are crucial for the large burst of IFN leading to induction of an anti-viral state. In these cells ATRA also up-regulates IRF-1 expression and binding to the RIG-I promoter. This leads to a ‘priming’ of the RIG-I promoter to respond rapidly to other stimuli. Retinoids have been implicated in the regulation of a number of ISGs including IRF-1 [11], [12], [13], [14], [15], [16], [17], [18], [19] and RIG-I [10]. Both of these genes have RAREs in their promoter regions. The proximity of the IRF-1 response element to possible RAREs in the RIG-I promoter suggests that there may be interaction between IRF-1 and RARs binding to the RIG-I promoter and this is currently under investigation. A second signal may be required to activate transcription in the U937 model, as has been observed for the Dif2 promoter [55].

Even in the absence of ATRA, MeV infection was able to induce some production of type I IFN in our cell culture models [9]. This likely occurs through detection by RIG-I early in infection, as the uncapped leader RNA of the virus is a known RIG-I ligand [22]. We hypothesize that IFN produced by these initially infected cells provides the second signal to the uninfected cells, allowing them to transcribe large amounts of RIG-I. In our U937 model, ATRA alone does not induce RIG-I mRNA but it can work in combination with IFNβ to induce high levels of RIG-I comparable to those seen with the combination of ATRA treatment and MeV infection (Figure 1A).

Measles-associated mortality and morbidity is correlated with both the infecting inoculum [56] and the extent of viral replication [57]. Early control of MeV replication may therefore influence the severity of disease. The ability of retinoids to induce RIG-I leading to the expression of type I IFN could contribute to the antiviral state and limit total body viral burden. We are currently investigating the effect of retinoids on other paramyxoviruses.

Our current and previous studies use a variety of cell culture and primary tissue, additionally; retinoids have been implicated in T lymphocyte proliferation and cytotoxicity [58], B cell proliferation [59] dendritic cell migration [60]. The combination of retinoids and MeV in these primary cells will be investigated in a small animal model. Our current studies do not address the timing of retinoid treatment during clinical MeV infection however. A small animal model is required to investigate the role of RIG-I during established MeV infection [61].

In conclusion, we have demonstrated that RIG-I expression is regulated by ATRA during MeV infection and is required for the anti-MeV state. We have also shown that IRF-1 is regulated by ATRA and plays a central role in mediating the anti-MeV effects of retinoids. IRF-1 is recruited to the RIG-I promoter under the influence of ATRA alone, and is required for the induction of RIG-I. In these models systems therefore, ATRA inhibits MeV replication through the RARα-dependent regulation of RIG-I and IRF-1 and via an IFN feedback loop.

## Materials and Methods

### Cells, reagents and viruses

All cell cultures were maintained at 37°C in a 5% CO_2_ humidified incubator. U937, NB4 and R4 cells were cultured as previously described [8]. The human hepatoma cell lines Huh7 and Huh7.5 (courtesy C. Richardson, Dalhousie University, Halifax, NS) were maintained in Dulbecco modified Eagle medium (Wisent, St-Bruno, QC) supplemented with 10% heat-inactivated fetal bovine serum (FBS; Wisent, St-Bruno, QC) and 0.1% gentamicin. HeLa (ATCC, #CCL-2) cells were maintained in Dulbecco modified Eagle medium (Wisent, St-Bruno, QC) supplemented with 10% heat-inactivated FBS (Wisent, St-Bruno, QC) and 1% penicillin/streptomyocin. All-trans retinoic acid (ATRA) (Sigma-Aldrich Fine Chemicals, ON) was kept as a stock solution at 10^−2^ M in 100% DMSO and further dilutions were performed in media. All retinoids were stored in opaque eppendorf tubes at −80°C and were handled in low-light conditions. The Chicago-1 MeV strain is a tissue culture-adapted genotype D3 virus (courtesy of W. Bellini, CDC, Atlanta, GA). MeV stock was grown by infecting Vero cells (ATCC, #CCL-81) at a multiplicity of infection (MOI) of 0.001 at 33°C in a Celligen Plus Bioreactor System (New Brunswick Scientific, Edison, NJ). Cell lines were infected with MeV and treated with ATRA as previously described [9] using the specific MOIs and time points indicated in the figure legends.

### Quantitative RT-PCR

RNA was extracted using Trizol (Invitrogen, Life Technologies) as per the manufacturer's instructions, and treated to remove possible genomic DNA contamination with Turbo DNAse (Ambion, Austin, TX). For experiments in which antibodies were used to block type I IFN signaling, an RNeasy Mini kit was used to extract RNA (Qiagen, Mississauga, ON). Equal quantities of RNA were reverse-transcribed into cDNA for qPCR analysis using random primers. FAM-labeled TaqMan primer-probe assays for the following genes were obtained from ABI (Foster City, CA): RIG-I, RARβ and IRF-1. The level of gene expression in untreated cells was used for calibration. Vic-labeled hGAPDH was used as the endogenous control.

### Western blotting

Cells were infected with MeV and/or treated with ATRA for indicated time periods washed in PBS and incubated in RIPA buffer on ice for 20 minutes (0.15 M NaCl, 0.05 M Tris-HCL, 1% Triton-X 100, 0.5% sodium deoxycholate, 0.1% SDS, 1 mM sodium orthovanadate). A protease inhibitor cocktail (Roche, Laval, QC) was diluted in RIPA buffer according to the manufacturer's instructions. The samples were pre-cleared, equal amounts of protein were separated by SDS-PAGE gel and transferred to PVDF membranes (Biorad, Hercules, CA). The membranes were incubated in 5% non-fat milk or 5% BSA for 1 hour and incubated overnight at 4°C with primary antibody. Primary antibodies used were against RIG-I (1/1000, courtesy of J. Hiscott, McGill University), IRF1 1/200, (Santa Cruz Biotechnology, CA, USA) and β-actin (1/10000, Sigma). Following overnight incubation, membranes were washed three times for 10 minutes in TBS/0.1% Tween, incubated with secondary antibody (1/10000, GE Healthcare) at room temperature for 60 minutes, and washed three times for 10 minutes. The peroxidase-conjugated secondary antibodies were developed using a chemiluminescence kit according to the manufacturer's instructions (GE Healthcare).

### Transwell and blocking antibody experiments

Transwell experiments (TW) were performed as previously described [9]. Briefly, cells infected at a low MOI are plated in 6-well plates. Transwell inserts were placed on top (0.02 µm pore membrane inserts, Nunc, Rochester, NY), and uninfected cells were placed in the inner chamber. The final overall MOI of the total well (inner plus outer chamber) was 0.01, and wells were either treated with DMSO or 1 µM ATRA. Control wells without membrane inserts were plated in the same manner, either with or without MeV, and with either DMSO or 1 µM ATRA. Cells were collected following 48 hours incubation and analyzed for gene expression by qPCR. Supernatants were also collected and used to treat fresh U937 cells. These fresh cells were pre-treated with anti-IFNAR2 blocking antibody (20 µg/µL, PBL Biomedical Laboratories, Piscataway, NJ) or isotype control antibody for one hour, and treated with these antibodies for the following 24 hours along with the conditioned media from the TW experiments. The blocking-antibody protocol was used to study the effect of IFN in the context of cells directly infected with MeV and/or treated with ATRA. These samples were analyzed by qPCR for the expression of RIG-I.

### Chromatin immunoprecipitation (ChIP)

ChIP assays were carried out using a modified Upstate Biotechnology protocol as previously described [52]. Briefly, 4×10^6^ cells were infected with MeV at an MOI of 0.1 and/or treated with 1 µM ATRA or DMSO for the indicated time periods. These cells were fixed in 1% formalin, lysed by sonication, and precleared with 80 µL of protein A agarose beads (Upstate, Millipore, Billerica, MA) for 1 hour in immunoprecipitation buffer (0.01% SDS, 1.1% Triton X-100, 1.2 mM EDTA, 16.7 mM Tris, 16.7 mM NaCl, 0.01 mg/ml aprotinin, 0.01 mg/ml leupeptin, 0.5 mM PMSF, 5 mM NaF). The samples were immunoprecipitated overnight with the appropriate antibody. Antibodies against the following proteins were used: IRF1 (5 µg Ab, Santa Cruz Biotechnology), RARα (2 µg Ab, Santa Cruz Biotechnology), Ac-Histone H3 (5 µg Ab, Upstate Millipore), and Pol II (5 µg Ab, Upstate Millipore,). Complexes bound to these antibodies were pulled down by 60 µL protein A agarose beads during a 4 hour incubation, and then washed in buffers of increasing stringency and eluted in elution buffer (1% SDS, 50 mM Tris, 10 mM EDTA). NaCl at 200 mM was used to reverse cross-linking at 65°C overnight. DNA extraction was performed using the QIAquick kit (QIAGEN, Mississauga, ON). qPCR analysis for the RIG-I promoter was performed using the following primers: forward (CAGCCGACGTGGGAGAACT), reverse (GCGCTAACGTTTAGACACAGTAAAAT). Input DNA was used as an endogenous control. Treatments were performed in duplicate, and qPCR amplifications were performed in triplicate.

### Luciferase assay

RIG-I promoter constructs (courtesy P. Fisher, Colombia Univeristy, New York, NY) were cloned into the pGL3-basic vector and mutated as described [24]. Two RIG-I constructs were utilized: a ‘full-length’ promoter construct (pRIGI-full) including -1902 bp to -1 bp and a site directed mutatgenesis of the IRF-binding site mutant of the full-length construct (pRIGI-IRFmut). HeLa cells seeded at 1.5×10^5^ cells/mL were transfected 12 hours later with 3 µg of luciferase expression construct and 0.5 µg of control Renilla construct using 3∶1 ratio of FuGENE 6 (Roche, Toronto, ON) as per the manufacturer's instructions. Samples were treated with 1 µM ATRA or DMSO or 1000 U/mL IFNß for 24 hours and analyzed for luciferase expression using the Dual Luciferase Assay System (Roche, Toronto, ON). Luciferase activity was normalized to Renilla expression and samples were analyzed in triplicate.

### Dominant negative transfection

Huh 7 cells were seeded at 1×10^5^cell/mL were infected 12 hours later with MeV MOI 0.01 and/or treated with 1 µM ATRA or DMSO. Post infection 3 µg of the dominant-negative RIG-I construct (RIG-IC) (gift from J. Hiscott) or empty vector were transfected using 3∶1 ratio of FuGENE 6 (Roche, Toronto, ON) as per the manufacturer's instructions. 48 hours post infection the cells and supernatants were quantified using plaque assay as previously described [8].

### RIG-I Over-expression transfection

Huh 7.5 cells were seeded at 1.5×105cell/mL were transfected with 3 µg of the RIG-I construct in a pcDNA3 plasmid (gift from J. Hiscott) or empty vector using a 3∶1 ratio of FuGENE 6 (Roche, Toronto, ON) as per the manufacturer's instructions. 18 hours post-transfection cells were infected with MeV MOI 0.01. 48 hours post infection the cells and supernatants were quantified using plaque assay as previously described [8].
